# A Biomechanical Comparison of Successful and Unsuccessful Snatch Attempts among Elite Male Weightlifters

**DOI:** 10.3390/sports7060151

**Published:** 2019-06-21

**Authors:** Hideyuki Nagao, Yasuyuki Kubo, Tempei Tsuno, Sho Kurosaka, Masato Muto

**Affiliations:** 1 Department of Sport Research, Japan Institute of Sports Sciences, 3-15-1, Nishigaoka, Kita-ku, Tokyo 115-0056, Japan; 2 Department of Sport Sciences, Japan Institute of Sports Sciences, 3-15-1, Nishigaoka, Kita-ku, Tokyo 115-0056, Japan; yasuyuki.kubo@jpnsport.go.jp (Y.K.); tempei.tsuno@jpnsport.go.jp (T.T.); sho.kurosaka@jpnsport.go.jp (S.K.); 3 Sports Medical Center, Japan Institute of Sports Sciences, 3-15-1, Nishigaoka, Kita-ku, Tokyo 115-0056; masato.muto@jpnsport.go.jp

**Keywords:** barbell trajectory, kinematics, kinetics, weightlifting, force, power

## Abstract

The success factor of the snatch has not been identified. Determining the success factors of the snatch among elite weightlifters might help to attain a successful snatch. This study aimed at clarifying the factors that lead to a successful snatch based on barbell trajectory among elite male weightlifters. Data were collected at the 2017 World and Junior World Weightlifting Championships. We digitized the barbell trajectory of the successful and unsuccessful snatch attempts of 61 lifters—an unsuccessful lift would be as a result of a frontward barbell drop—and calculated the kinematic and kinetic parameters of the barbell. No significant difference was found in the barbell maximum height (Dy1) between the successful and unsuccessful lifts. The amount of backward displacement of the barbell in the second pull phase to the catch position (DxL) of the successful lift was significantly larger than that of the unsuccessful lift (successful: 0.11 ± 0.05 m; unsuccessful: 0.10 ± 0.06 m; *p* < 0.01; d = 0.278). The barbell drop distance in the catch phase (Dy3) of the successful lift was significantly smaller than that in the unsuccessful lift (successful: 0.17 ± 0.04 m; unsuccessful: 0.18 ± 0.04 m; *p* < 0.001, d = 0.361). These results suggest that DxL and Dy3 are factors leading to a successful snatch lift, but not Dy1. The relative position in the sagittal axis of the barbell and the lifter in the catch position, and catching the barbell when its momentum was low, are important in order to achieve a successful snatch.

## 1. Introduction

Olympic-style weightlifting consists of the snatch and the clean and jerk. Athletes are allowed three attempts in each lift, and are ranked according to the score of the best weight lifted. Lifters try a relatively low weight instead of the maximum weight in the first attempt, because the barbell is loaded progressively. A lifter cannot attempt to lift a weight that is less than the weight they previously tried. Therefore, many unsuccessful attempts lead to inferior results. In the Olympic Games, if a lifter is unsuccessful in all snatch attempts, that lifter is not allowed to try any clean and jerks, and therefore achieves no total. Thus, increasing the success rate of the snatch is important for improving the weightlifting competition result.

Some weightlifters can achieve a successful snatch lift with a load equal to or greater than a previously unsuccessful lift. In other words, an unsuccessful lift occurs not only when the lifted weight exceeds the lifters’ weight maximum, but when technical factors are at play. Studies on snatch performance have focused mainly on the differences between superior and inferior lifters, or the difference between the lifted barbell weight with respect to barbell kinematics, kinetics, and joint kinematics [1,2,3]. Measurement findings of these studies revealed that the skill of the second pull phase, including lower limb joint kinematics and barbell kinematics, is important for a successful heavy snatch lift. A few studies have examined the differences between the successful and unsuccessful snatch. Stone et al. [4] compared the barbell kinematics and kinetics of 43 men with successful and unsuccessful snatch lifts in U.S. National Weightlifting Championships, and the concurrent North American, Central American, and Caribbean Island Championships. In that study, no clear difference was found between successful and unsuccessful snatch lifts with regard to the amount of displacement, the velocity of the barbell, or the force and power applied to the barbell. Those findings suggested that no single variable could explain why one snatch was successful and another was not. Thus, success in weightlifting is multifactorial. However, that study included cases wherein the weights were different in the successful and unsuccessful lifts. Additionally, they did not clearly specify the cause of the failure in an unsuccessful attempt. The main type of unsuccessful snatch lift is a drop of the barbell in front of or behind the lifter. Depending on how that lift was unsuccessful, it is expected that the factors leading to success or failure will be different. Gourgoulis et al. [5] studied the biomechanical differences between successful and unsuccessful snatch lifts in seven men in the 69, 77, and 85 kg weight categories in the adult Greek national weightlifting team who experienced a frontward drop of the barbell. The authors reported no significant difference in the barbell maximum height, amount of forward and backward displacement, or velocity in each of the phases. Furthermore, there was no significant difference in the joint kinematics or in the relative position of the foot, knee, and shoulder with reference to the barbell’s position. The only statistically significant difference was in the angle of the barbell’s resultant linear acceleration vector, relative to the vertical axis between the successful and unsuccessful lifts observed during the first pull phase. However, that study was limited by their small sample size, which restricted the generalizability of the results [6].

To clarify the general unsuccessful factor of the snatch, it is necessary to use a large sample size of the same lifter’s successful and unsuccessful snatch lifts with the same weights, and to classify the unsuccessful snatch lift types. Determining the exact success factors of the snatch in elite weightlifters might help to improve the probability of a successful snatch. The purpose of this study is to clarify the success factors of the snatch based on barbell trajectory among elite male weightlifters, in case of an unsuccessful lift as a result of a frontward barbell drop.

## 2. Materials and Methods

### 2.1. Weightlifters

To examine the differences in the snatch between successful and unsuccessful lifts, barbell trajectory data were collected during the 2017 International Weightlifting Federation (IWF) World Championship, and the IWF Junior World Championship. The data were then quantified by video analysis. Permission for filming for the purpose of the study was granted by the IWF. Our study has obtained the approval from the institutional review board at the Japan Institute of Sport Sciences.

The snatch attempts were recorded using a digital video camera (HDR-CX670, SONY, Tokyo, Japan) operating at 60 Hz, with a shutter speed set at 1/500 sec. The camera was placed approximately 20 m from the lifting platform and almost perpendicular to the left side of the sagittal plane. In this study, the data included successful and unsuccessful snatch lifts achieved at the same weights in the same lifter— an unsuccessful lift was attributed to a frontward barbell drop. If there were two unsuccessful lifts at the same weight, the first unsuccessful lift was used for the study data. The categories (junior or senior), weight categories, and barbell weights are listed in Table 1. Sixty-one lifters had successful and unsuccessful snatch lifts achieved at the same weights, with frontward drops in 122 total snatch lifts. Some of the recorded snatch lift motions were not analyzed, because the lifter and the lift could not be viewed clearly because of obstacles passing in front of the camera during recording. 

### 2.2. Procedures

To obtain the real-space two-dimensional position coordinates of the barbell trajectory in the sagittal plane, the left end of the barbell was digitized to obtain the position coordinates in the camera space. The speeded-up robust features method [7] was employed for automatic digitizing. According to a previous study [8], the barbell plate diameter (0.45 m) was used as the reference for calibrating the barbell’s real-space position coordinates from the camera-space position coordinates. The coordinate origin was set at the start position of the barbell. With respect to the axes, the positive and negative values of the *x*-axis represent the forward and backward motions of the lifter, respectively, and the positive values of the *y*-axis represent the vertical upward motions of the lifter. There was no guarantee that the barbell movement would be symmetric. However, the left barbell end was analyzed as the representative point of the barbell’s trajectory in present study.

**Figure 1:**
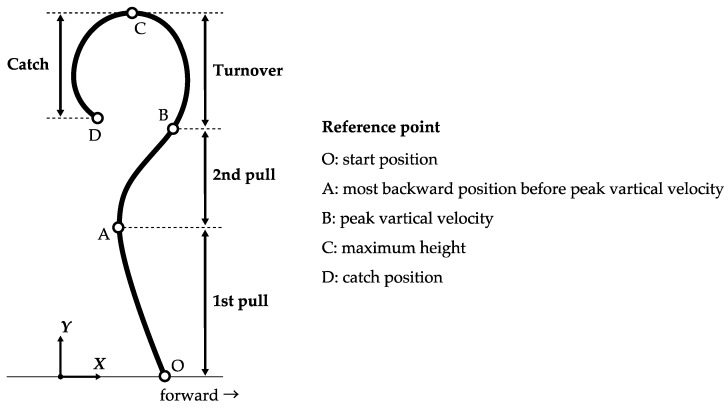
Phases and reference points of the snatch lift. The coordinate origin O was set at the start position. The direction of the lifter’s line of sight was set at the forward direction.

The coordinate values were filtered digitally using a Butterworth fourth-order low-pass filter with a cutoff frequency of 4 Hz, as in previous research studies [9,10]. To obtain the data on the barbell kinematics and kinetics, the snatch lift phases were defined according to the barbell trajectory (Figure 1). The first pull phase covered from the start position to the most backward position, before reaching the peak vertical velocity of the barbell. The second pull phase was the end of the first pull phase to the peak vertical velocity of the barbell. The turnover phase was the end of the second pull phase to the maximum height of the barbell. The catch phase started from the end of the turnover phase to the catch position. The “start position” was defined as the time when the *y*-axis component of the barbell position (barbell height) was ≥ 0.225 m, and the *y*-axis component of the barbell velocity was ≥0.01 m/s. The “catch position” was defined as the time when the *y*-axis component of the barbell velocity was closest to 0 m/s after the height of the barbell reached the maximum.

**Figure 2:**
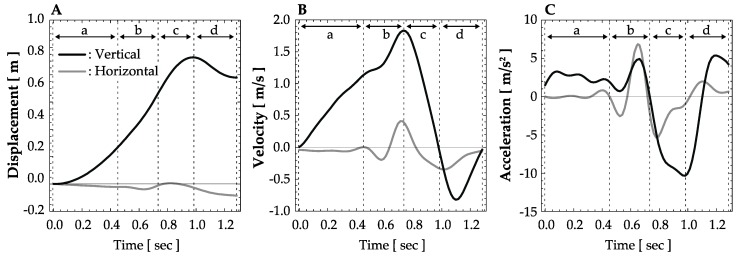
An example of barbell displacement, velocity, and acceleration. (**a**) First pull phase; (**b**) second pull phase; (**c**) turnover phase; (**d**) catch phase. (**A**) displacement; (**B**) velocity; (**C**) acceleration.

Examples of the *y-* and *x*-axis displacement components, velocity, and acceleration calculated from the barbell trajectory in the snatch lift are shown in Figure 2. The barbell kinematics and kinetics parameters were calculated based on the abovementioned calculations, and their definitions are shown in Table 2. 

**Figure 3:**
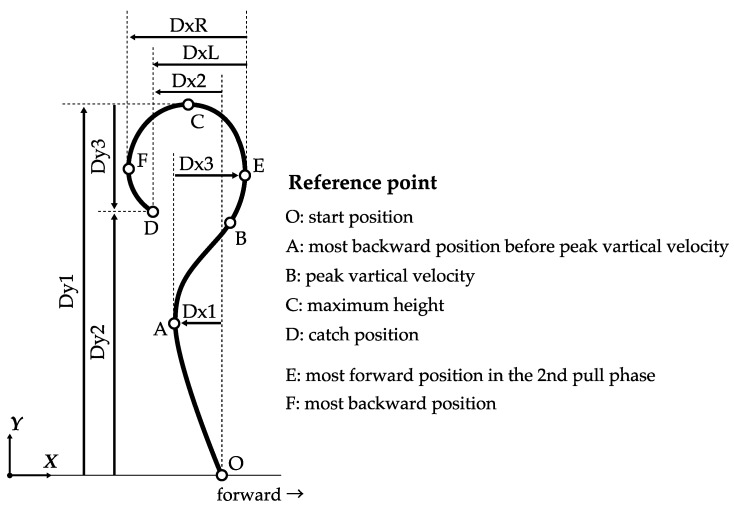
Experimental variables in barbell kinematics. The direction of the lifter’s line of sight was set at the forward direction. The definition of Dx1, Dx2, Dx3, DxL, DxR, Dy1, Dy2 and Dy3 are shown in Table 2.

The kinematics parameters of the displacement of the barbell are shown in Figure 3. The force applied to the barbell was calculated based on the methods of a previous study [2]. The barbell horizontal force was calculated as the product of the horizontal acceleration of the barbell and barbell mass. The barbell vertical force was calculated by adding the product of the barbell mass and 9.8 to the product of the vertical acceleration and barbell mass.

### 2.3. Statistical Analyses

All of the data were presented as means ± standard deviation (SD). The Kolmogorov–Smirnov test was used to test the normality of the distributions. Paired *t*-tests were used to compare all of the barbell variables between the successful and unsuccessful snatch lifts. The magnitude of the differences was determined via the calculation of Cohen’s *d*-effect size. The magnitudes of the effect sizes were interpreted as small (~0.2), medium (~0.5), and large (~0.8) [11]. The level of significance was set at *p* < 0.05 for all of the statistical tests performed.

## 3. Results 

In the 2017 IWF World Championship and Junior World Championship, the success rate and failure rate of snatch in men were 61.2% (574 attempts) and 38.8% (363 attempts), respectively. The highest percentage of unsuccessful lift types was observed in the frontward barbell drop (65.8%; 239 attempts), followed by the backward barbell drop (25.1%; 91 attempts). Other unsuccessful lift types included the press-out and quit lift before second pull (9.1%; 33 attempts). 

Barbell parameters with statistically significant differences, with a small effect size were found between successful and unsuccessful snatch lifts (Table 3). The difference between successful and unsuccessful snatch was characterized by Dy3, pFy_2nd, DxL, and DxR.

## 4. Discussion

### 4.1. Vertical Barbell Kinematics and Kinetics 

The results in this study reveal no significant difference between the successful and unsuccessful lifts in the barbell parameters of Dy1, Dy2, and pVy+_2nd. These results indicate that the maximum barbell height was high enough in the unsuccessful snatches in the elite lifters. Harbili et al. [1] compared the heaviest snatch lift and the next heaviest lift in nine elite male adolescent weightlifters, and they reported that the barbell maximum height (Dy1) and peak vertical velocity in the second pull phase (pVy+_2nd) were significantly lower in the heaviest lift than in the next heaviest lift. Isaka et al. [12] demonstrated that the relative maximum height of the barbell (Dy1/body height) and the relative catch position (Dy2/body height) for the best lifter were smaller, and the peak vertical velocity of the barbell was smaller than that for the lower-ranking lifters in each weight category. These previous studies suggested that the superior lifter has a technique that allows them to catch the barbell at a low position, even if the peak velocity of the barbell is small and its maximum height is low. The higher the barbell is elevated off the ground, the more time there is for the lifter to move under the barbell to catch it. However, from the results of this study, a large amount of barbell elevation is not essential for a superior snatch technique. The results of this study support the findings of Gourgoulis et al. [5], who reported no significant difference between Dy1 and the vertical velocity-related variables in the successful and unsuccessful snatch lifts with frontward barbell drops. It suggests that the maximum barbell height does not determine the success or failure of the snatch lift. Furthermore, no significant differences were found in the pPy_1st and pPy_2nd between the successful and unsuccessful snatch lifts. This result is not surprising, because there were no significant differences in the velocity-related variables. Furthermore, this analysis was a within-subject analysis; therefore, the work in the first pull and second pull phases were considered almost the same. Gourgoulis et al. [5] also observed no significant difference in the work and power applied to the barbell in the first pull and second pull phases. Therefore, pPy_1st and pPy_2nd may not be direct causes of success or failure in the snatch lift.

The peak vertical force applied to the barbell in the second pull phase (pFy_2nd) was significantly larger in the successful lift than in the unsuccessful lift. In contrast, Kipp et al. [13] showed a significant positive correlation between the body mass-normalized weightlifting in the snatch and the vertical acceleration of the barbell, and pointed out its importance in the snatch. From the perspective of the successful or unsuccessful snatch lift, the lifter’s posture and motion, which exert large forces, appear to influence the subsequent motions and promote an appropriate catch position. The difference in the timing of the peak velocity and peak force may be a reason for the significant difference in the peak force, despite no significant difference in peak power. Furthermore, a significant difference was found in the barbell drop distance (Dy3) between the successful and unsuccessful snatch lifts. The drop distance in the catch phase has emerged as a significant indicator of efficiency in the snatch technique [12,14]. In successful lifts, it can be inferred that the lifter would catch the barbell when its momentum is low, by decreasing the drop distance. Therefore, it is suggested that the barbell drop distance determined the success or failure of the snatch lift.

### 4.2. Horizontal Barbell Kinematics and Kinetics

This study revealed that when dropping the barbell in front in an unsuccessful lift, DxL and DxR were significantly larger in the successful lift than those in the unsuccessful lift. These results suggested that DxL and DxR are factors of a successful snatch. To catch the barbell, the lifter has to move the barbell and bring it to the proper position, and the catch position involves keeping the arms extended and overhead in a squatting position. The relative positions of the barbell and the lifter are determined by the amount of forward and backward displacement of the barbell, the forward and backward displacement of the lifter, and the posture of catch position. The results of this study suggested that DxL and DxR affect the relative positions of the barbell and the lifter, and influence the success or failure of the lift. Isaka et al. [12] reported that the barbell should move along the vertical reference line of the barbell’s start position, with minimal horizontal displacement. This would reduce the horizontal work required for an effective lifting technique. In contrast, Stone et al. [4] suggested that lifts with a backward displacement of the barbell (DxL) are also regarded as a positive technique for producing force. From the perspective of the successful snatch lift, the amount of backward barbell displacement from the second pull phase to the catch phase is in the appropriate range to catch the barbell, with neither a maximization nor minimization of either parameter. 

Gourgoulis et al. [5] suggested that skilled weightlifters showed a stable pattern in their barbell movement, regardless of the final outcome of the lift. However, we revealed several significant differences in the barbell kinematics and kinetics. This study obtained results different from those in previous studies, through the analysis of a relatively large sample size of lifters. 

### 4.3. Limitations

This study compared successful and unsuccessful snatch lifts using the same weights; the unsuccessful lift was due to the frontward barbell drop only. Hence, the success factors of snatch presented in this study may not be applicable to other types of unsuccessful snatch, such as the backward barbell drop. Moreover, whether the success factor of the snatch using weights exceeding the maximum, based on the weight of the lifters, is consistent with our results remains unclear.

## 5. Conclusions

The maximum height of the barbell (Dy1) was not significantly different between the successful and unsuccessful lifts. Therefore, the maximum height of the barbell may not be considered as a factor in the success or failure of the snatch. In contrast, the amount of backward displacement of the barbell from the most forward position in the second pull phase to the catch phase (DxL), and the amount of backward displacement of the barbell from the most forward position in the second pull phase to the most backward position in the catch phase (DxR) were significantly larger in the successful lifts than those in the unsuccessful lifts. We concluded that DxL and DxR are successful factors in the snatch lift. That is, the relative position of the barbell and the lifter in the catch position are important in order to be able to catch the barbell. The drop distance of the barbell in the catch phase (Dy3) was significantly smaller in the successful lifts than in the unsuccessful lifts. Small drop distances could increase the chances of success, because of the advantages conferred in reducing the drop distance, which reduces the momentum of the barbell. It is advantageous to set that velocity to zero. Therefore, the barbell drop distance determines the success or failure of the snatch lift. In addition, in case of an unsuccessful lift because of a frontward barbell drop, paying attention to the barbell backward displacement and drop distance may increase the chances of success in the next snatch lift.

## Figures and Tables

**Table 1:** Characteristics of the weightlifters and the barbell.

Category	Weight Category	Snatch [kg]	Category	Weight Category	Snatch [kg]
junior	56	98	junior	85	142
senior	56	105	junior	85	145
senior	56	111	junior	85	145
senior	56	112	junior	85	145
junior	62	105	junior	85	147
junior	62	108	junior	85	150
junior	62	110	senior	85	140
junior	62	116	senior	85	145
junior	62	127	senior	85	150
senior	62	117	senior	85	155
senior	62	122	senior	85	160
senior	62	126	junior	94	147
senior	62	130	junior	94	151
senior	62	130	senior	94	150
senior	62	130	senior	94	155
senior	69	124	senior	94	157
senior	69	132	senior	94	166
senior	69	132	junior	105	121
senior	69	135	junior	105	160
senior	69	135	senior	105	150
junior	77	131	senior	105	150
junior	77	131	senior	105	165
junior	77	134	senior	105	176
junior	77	137	senior	105	177
junior	77	140	senior	+105	175
junior	77	149	senior	+105	184
junior	77	152	senior	+105	185
senior	77	133	senior	+105	203
senior	77	145		Mean	142.0
senior	77	148		S.D.	± 21.2
senior	77	150	senior: n = 38		
senior	77	150	junior: n = 23		
senior	77	158	Total: n = 61		

**Table 2:** Experimental variables in barbell kinematics and kinetics.

Symbol	Unit	Definition
**Barbell vertical direction variable**		
Dy1	[m]	Start position to maximum height
Dy2	[m]	Start position to the catch position
Dy3	[m]	Maximum height to the catch position (drop distance)
pVy+_1st	[m/s]	Maximum vertical linear velocity in the 1st pull phase
pVy+_2nd	[m/s]	Maximum vertical linear velocity in the 2nd pull phase
pVy-	[m/s]	Minimum vertical linear velocity in the catch phase (drop velocity)
pFy_1st	[N]	Maximum vertical linear force in the 1st pull phase
pFy_2nd	[N]	Maximum vertical linear force in the 2nd pull phase
pPy_1st	[W]	Maximum vertical linear power in the 1st pull phase
pPy_2nd	[W]	Maximum vertical linear power in the 2nd pull phase
pFy%height	[%]	Height of peak vertical force position normalized by the maximum height
**Barbell horizontal direction variable**		
Dx1	[m]	Start position to the most backward position before the turnover phase
Dx2	[m]	Start position to the catch position
Dx3	[m]	Most backward position before the turnover phase to the most forward position
DxL	[m]	Most forward position in the 2nd pull phase to the catch position
DxR	[m]	Most forward position in the 2nd pull phase to the most backward position
pVx+	[m/s]	Maximum horizontal linear velocity in the forward direction
pVx-	[m/s]	Maximum horizontal linear velocity in the backward direction
pFx+	[N]	Maximum horizontal linear force in the forward direction
pFx-	[N]	Maximum horizontal linear force in the backward direction

**Table 3:** Barbell kinematics and kinetics in successful and unsuccessful lifts. SD—standard deviation.

Variables	Unit	Successful			Unsuccessful			*p*-Value		Effect Size	
Dy1	[m]	1.31	±	0.09	1.32	±	0.09	0.280		0.017	
Dy2	[m]	1.15	±	0.09	1.13	±	0.09	0.001		0.159	
Dy3	[m]	0.17	±	0.04	0.18	±	0.04	0.000	**	0.361	†
pVy+_1st	[m/s]	1.00	±	0.12	1.01	±	0.13	0.130		0.090	
pVy+_2nd	[m/s]	1.93	±	0.13	1.93	±	0.14	0.560		0.010	
pVy-	[m/s]	‒0.81	±	0.13	‒0.80	±	0.13	0.473		0.006	
pFy_1st	[N]	1962	±	365	1959	±	380	0.405		0.007	
pFy_2nd	[N]	2131	±	459	2047	±	366	0.000	**	0.202	†
pPy_1st	[W]	373	±	157	382	±	156	0.304		0.056	
pPy_2nd	[W]	1198	±	504	1205	±	467	0.438		0.016	
pFy%height	[%]	60.4	±	11.4	62.6	±	10.2	0.129		0.203	
Dx1	[m]	0.07	±	0.03	0.06	±	0.03	0.185		0.063	
Dx2	[m]	0.12	±	0.09	0.11	±	0.09	0.021		0.169	
Dx3	[m]	0.06	±	0.03	0.06	±	0.03	0.187		0.054	
DxL	[m]	0.11	±	0.05	0.10	±	0.06	0.005	*	0.278	†
DxR	[m]	0.13	±	0.07	0.11	±	0.06	0.000	**	0.254	†
pVx+	[m/s]	0.47	±	0.18	0.47	±	0.17	0.408		0.014	
pVx-	[m/s]	‒0.39	±	0.10	‒0.38	±	0.09	0.125		0.089	
pFx+	[N]	976	±	428	991	±	420	0.191		0.036	
pFx-	[N]	‒773	±	243	‒813	±	265	0.084		0.083	
mean±S.D.											

note: *: *p* < 0.05, **: p < 0.01, ***: p < 0.001, †: d > 0.20.
